# Innovation in the Breeding of Common Bean Through a Combined Approach of *in vitro* Regeneration and Machine Learning Algorithms

**DOI:** 10.3389/fgene.2022.897696

**Published:** 2022-08-24

**Authors:** Muhammad Aasim, Ramazan Katirci, Faheem Shehzad Baloch, Zemran Mustafa, Allah Bakhsh, Muhammad Azhar Nadeem, Seyid Amjad Ali, Rüştü Hatipoğlu, Vahdettin Çiftçi, Ephrem Habyarimana, Tolga Karaköy, Yong Suk Chung

**Affiliations:** ^1^ Faculty of Agricultural Sciences and Technologies, Sivas University of Science and Technology, Sivas, Turkey; ^2^ Department of Metallurgical and Materials Engineering, Faculty of Engineering and Natural Sciences, Sivas University of Science and Technology, Sivas, Turkey; ^3^ Department of Plant Production and Technologies, Faculty of Agricultural Science and Technologies, Sivas University of Science and Technology, Sivas, Turkey; ^4^ Center of Excellence in Molecular Biology, University of the Punjab, Lahore, Pakistan; ^5^ Department of Information Systems and Technologies, Bilkent University, Ankara, Turkey; ^6^ Department of Field Crops, Faculty of Agriculture, University of Çukurova, Adana, Turkey; ^7^ Department of Field Crops, Faculty of Agriculture, Bolu Abant Izzet Baysal University, Bolu, Turkey; ^8^ International Crops Research Institute for the Semi-Arid Tropics, Patancheru, India; ^9^ Department of Plant Resources and Environment, Jeju National University, Jeju, South Korea

**Keywords:** machine learning algorithms, artificial neural network, *in vitro* regeneration, plumular apices, coefficient of determination, mean squared error

## Abstract

Common bean is considered a recalcitrant crop for *in vitro* regeneration and needs a repeatable and efficient *in vitro* regeneration protocol for its improvement through biotechnological approaches. In this study, the establishment of efficient and reproducible *in vitro* regeneration followed by predicting and optimizing through machine learning (ML) models, such as artificial neural network algorithms, was performed. Mature embryos of common bean were pretreated with 5, 10, and 20 mg/L benzylaminopurine (BAP) for 20 days followed by isolation of plumular apice for *in vitro* regeneration and cultured on a post-treatment medium containing 0.25, 0.50, 1.0, and 1.50 mg/L BAP for 8 weeks. Plumular apice explants pretreated with 20 mg/L BAP exerted a negative impact and resulted in minimum shoot regeneration frequency and shoot count, but produced longer shoots. All output variables (shoot regeneration frequency, shoot counts, and shoot length) increased significantly with the enhancement of BAP concentration in the post-treatment medium. Interaction of the pretreatment × post-treatment medium revealed the need for a specific combination for inducing a high shoot regeneration frequency. Higher shoot count and shoot length were achieved from the interaction of 5 mg/L BAP × 1.00 mg/L BAP followed by 10 mg/L BAP × 1.50 mg/L BAP and 20 mg/L BAP × 1.50 mg/L BAP. The evaluation of data through ML models revealed that *R* ^*2*^ values ranged from 0.32 to 0.58 (regeneration), 0.01 to 0.22 (shoot counts), and 0.18 to 0.48 (shoot length). On the other hand, the mean squared error values ranged from 0.0596 to 0.0965 for shoot regeneration, 0.0327 to 0.0412 for shoot count, and 0.0258 to 0.0404 for shoot length from all ML models. Among the utilized models, the multilayer perceptron model provided a better prediction and optimization for all output variables, compared to other models. The achieved results can be employed for the prediction and optimization of plant tissue culture protocols used for biotechnological approaches in a breeding program of common beans.

## Introduction

Grain legumes are an important pillar of the agricultural system, are considered a vital source of high-quality protein for food and fodder, and play a significant role in sustainable cropping systems (Vanlauwe et al., 2019). Common bean (*Phaseolus vulgaris L*.) is an important grain legume crop and is mostly used worldwide for its pods and palatable seeds (Nadeem et al., 2020a). Common bean contains good concentrations of high-quality protein, minerals particularly zinc and iron, vitamins, and antioxidants and is considered a “grain of hope” for the impoverished community. Common bean was originated in Mesoamerica (Bitocchi et al., 2012) and its domestication in Andean and Mesoamerican regions resulted in the formation of two unique gene pools: Andean gene pool and Mesoamerican gene pool (Kami et al., 1995; Mamidi et al., 2013; Asfaw et al., 2017; Blair et al., 2018; Campa et al., 2018). Common bean is considered one of the most varied legume crops by reflecting variations in its growth habit, plant height, pods, maturity, seed weight and size (Yeken et al., 2019; Nadeem et al., 2020b).

Climate change is becoming a serious threat to agriculture, and various biotic (pathogens and insects) or abiotic (drought and edaphic) factors are contributing significantly to the global common bean production loss (Castillo et al., 2015). Keeping these in view, scientists are trying to develop climate-resilient common bean cultivars having improved agronomic and nutritional traits. The mentioned target can be achieved by the application of modern biotechnological techniques and for that reason, optimization of the *in vitro* plant tissue culture technique for whole plant regeneration is highly demanding. To date, a reasonable number of *in vitro* regeneration protocols have been established and documented. *In vitro* regeneration of common bean is an arduous task due to its recalcitrant nature, genotype dependence, lack of reproducibility, low shoot counts with stunted growth, rooting, and acclimatization. Hence, there is always a need to develop a new, efficient, and repeatable protocol for the application of biotechnological techniques to produce elite cultivars, especially for recalcitrant crops (Aasim et al., 2013). To achieve the objective, selection of potent explants with a high regeneration protocol is highly significant. Considering this, a novel explant “plumular apices” and an *in vitro* regeneration protocol of pretreatment of explants with high benzylaminopurine (BAP) concentration was employed for common bean. Pretreatment is the process of treating seeds or explants with variable stimulants like cytokinins at low to high doses for a certain period, followed by culturing the explants on a post-treatment medium, supplemented with low plant growth regulators (PGRs) or without any PGRs (Özkan and Aasim, 2019).

Conventional plant breeding methodologies include the assessment and classification of genetic diversity, yield component analysis, yield stability analysis, enhanced tolerance to stresses, and hybrid breeding programs. On the other hand, *in vitro* micropropagation, doubled haploid production, artificial polyploidy induction and *Agrobacterium*-mediated gene transformation techniques are considered *in vitro*-based biotechnological breeding methodologies (Niazian and Niedbala, 2020). In plant tissue culture studies, the impacts of input (uni or multi) factors on the regeneration potential (outputs) of desired plants are studied. In general, classical statistical techniques have been employed for analyzing and interpreting the output variables. These techniques are generally based on variance analysis and linear regression models for estimating the correlation between input (independent) and output (dependent) variables. Although these approaches are highly effective, lack of efficacy of complex and nonlinear inputs (Hesami and Jones, 2021; Earl et al., 2021) and high probability (Abbasi et al., 2016; Jamshidi et al., 2016; Farhadi et al., 2020) are the major concerns in plant tissue culture studies due to the sensitivity. These types of issues can be overcome by modern high throughput technologies like machine learning (ML) and artificial neural network (ANN) models for testing and optimizing the output variables concerning the input parameters. Although the application of ML and ANN models in plant sciences specifically in the area of plant tissue culture is in its early stages, it is successfully documented for different aspects of plant tissue culture ranging from *in vitro* sterilization to *in vitro* regeneration and from *in vitro* callogenesis to secondary metabolite production (Hesami et al., 2020a; García-Pérez et al., 2020; Hameg et al., 2020; Hesami & Jones, 2020; Niazian and Niedbala, 2020; Pepe et al., 2021; Salehi et al., 2021; Aasim et al., 2022). In these studies, researchers employed different ML algorithms, and the selection of specific ML models is generally based on the expertise and target set in the study. These data-driven models are highly efficient to parse and interpret different types of datasets (non-normal, nonlinear, and nondeterministic unpredictable data) by using all spectral data along with avoiding irrelevant spectral bands and multicollinearity (Salehi et al., 2020). In this study, an *in vitro* regeneration protocol of common bean was established using novel plumular apice explants. The results regarding output variables were analyzed and interpreted, and input variables were predicted by response surface methodology (RSM). In addition, the results for the output variables were validated using different ML algorithms (support vector regression—SVR, Gaussian process regression—GPR, XGBoost regression—XGBoost, and random forest regression—RF) and an ANN-based multilayer perceptron (MLP) regression model. The performance was evaluated by tabulating the *R* ^*2*^ and the mean squared error (MSE) metric values for each model (Hesami et al., 2019; Kirtis et al., 2022). The results achieved in this study will open a new window to evaluate the efficiency of the plant tissue culture protocols that are predominantly developed for breeding purposes.

## Materials and Methods

### *In vitro* Regeneration

**FIGURE 1:**
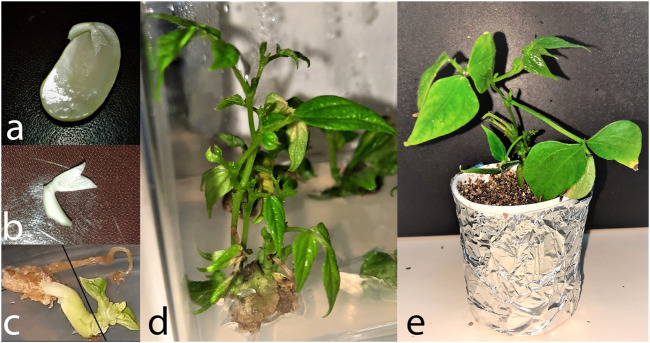
*In vitro* regeneration and rooting of common bean Cv. Karacaşehir 90 **(A)** sterilized seed with the intact embryo, **(B)** isolated embryo ready for inoculating on the pretreatment medium, **(C)** pretreated mature embryo used for isolating the plumular apice explant, **(D)** multiple shoot induction from the plumular apice explant, and **(E)** acclimatized plant in a pot containing vermiculite.

The commercial common bean cultivar “Karacaşehir-90” was selected for this study as the plant material. Manually selected uniform seeds were surface sterilized with 3.5% (w/v) NaOCl for 15 min. Thereafter, seeds were continuously rinsed with sterilized dH_2_O water for 5–7 min and this process was repeated thrice to remove the traces of NaOCl. Seeds (Figure 1A) were awaited for 24 h in dH_2_O, followed by isolation of mature embryos (Figure 1B) under aseptic conditions. A two-step experiment was designed for this research. At first, mature embryos isolated from sterilized seeds were inoculated on MS (Murashige and Skoog, 1962) media supplemented with 5, 10, and 20 mg/L BAP (pretreatment medium) for 20 days. In the second step, plumular apice explants (Figure 1C) were carefully isolated from pretreated mature explants, followed by inoculation on MS media supplemented with low BAP (0.25, 0.50, 1.00, and 1.50 mg L^−1^) concentrations (post-treatment medium). The explants were cultured for 8 weeks on the post-treatment medium. Four different concentrations (0.25, 0.50, 1.0, and 1.50 mg L^−1^) of indole-3-butyric acid (IBA) were used for *in vitro* rooting. For acclimatization, rooted plantlets were transferred to pots filled with vermiculite, wrapped in a polyethylene bag, and placed in the growth room.

The basal media used for pretreatment, post-treatment, and rooting were prepared by adding MS (4.4 g/L), commercial sugar (30 g/L), and polyvinyl proline (25 mg L^−1^). The pH of all media was adjusted to ∼5.8 with the aid of 1N HCl or 1N NaOH. The medium was gelled with agar (6.5 g/L) and autoclaved at 121°C for 20 min. All experiments were carried out in the growth room at 24 ± 2°C and 16-h light photoperiod, equipped with white light-emitting diodes at approximately 2000 LUX. All chemicals used in this study were procured from Duchefa (MS, BAP, IBA, and agar) and Sigma-Aldrich (polyvinyl proline).

### Response Surface Methodology

The RSM approach was used to model and optimize the selected responses to changing variables and graphical representation of the results. RSM generates continuous multivariable predictions represented as quadratic surfaces, allowing the prediction of optimal values in three-dimensional space. Pretreatment, post-treatment, and their interactive effect values were used as input variables. On the other hand, regeneration frequency (%), shoot count, and shoot length (cm) were used for the response surface calculations. The degree of predicted mathematical model compliance to obtained values was expressed as *R* ^*2*^ fit values. All RSM data analyses, such as analysis of variance, regression, and generation of quadratic polynomial surface equations, graphics and optimal value predictions, were conducted using Minitab v20.4 statistical software.

### Modeling Procedures

In this study, interactions of pretreatment (5, 10, and 20 mg/L BAP) × post-treatment BAP doses (0.25, 0.50, 1.0, and 1.50 mg/L) were used as input variables, whereas, *in vitro* regeneration frequency, shoot count, and shoot length were measured as the output variables. ML algorithms of SVR (Hesami et al., 2020b; Katirci and Takci, 2021), GPR (Hu et al., 2019), XGBoost (Chen and Guestrin, 2016), RF (Aggarwal, 2018), and MLP neural network (Silva et al., 2019) were utilized to train and test the model. The performance of the model was assessed using leave-one-out cross-validation (Sammut and Webb, 2011). The hyperparameters of the ML models were optimized using the grid search technique to find the best model. The open-source Python language (Van Rossum and Drake, 1994) was used to code algorithms using the sklearn library (Pedregosa et al., 2011). MLP, SVR, GP, XGBoost, and RF algorithms were used to predict the outputs. The model performance was evaluated by calculating *R* ^*2*^ (coefficient of determination) and MSE values (Hesami et al., 2019), which are presented in Eqs. 1 and 2.

$$R^{2}=1-\frac{\sum_{i=1}^{n}{\left(Y_{i}-{\hat{Y}}_{i}\right)}^{2}}{\sum_{i=1}^{n}{\left(Y_{i}-\tilde{Y}\right)}^{2}}$$ (1)

$$MSE=\frac{1}{n}\sum_{i=1}^{n}{\left(Y_{i}-{\hat{Y}}_{i}\right)}^{2}$$ (2)

$Y_{i}$ represents the measured values, ${\hat{Y}}_{i}$ indicates the predicted values, $\tilde{Y}$ denotes the mean of the measured values, and *n* is the count of samples.

The dataset that is used in plant tissue culture studies may not be linear; hence, nonlinear regression models are essential like the SVR model (Drucker, 1997) as expressed in Eq. 3. In SVR, the output *y* is a real number and it can be used for nonlinear variables.

y=wφ(x)+b (3)

In the above equation, *b* depicts the bias, *w* represents the weight, and the elevated features space is presented as φ(x) , which defines the nonlinearity of input *x*. In the SVR model, the predicted variable is placed between the upper and lower limit values to minimize the risk. In case the data exceeds these limits, it is set between these values (Smola & Schölkopf, 2004). The kernels of “linear”, “poly”, “radial basis function (rbf)”, “sigmoid”, and “precomputed” are present in the SVR model. Among these, the RBF kernel is the most widely used.

The GPR model is another nonparametric supervised learning method that is used mainly to perform Bayesian nonlinear regression and classification tasks. It is a powerful ML algorithm that uses the Gaussian probability density function. The GPR works efficiently with a small dataset, with more accuracy, ease of calculation, and consistency.

The approach is presented in Eq. 4 for each input *x* and output *y* produced by this function.

$$y_{i}=f\left(x_{i}\right)+\varepsilon$$ (4)

Extreme gradient boosting (XGBoost) is a decision-tree-based ensemble ML algorithm that uses a gradient boosting framework that can be used for both regression and classification problems (Chen and Guestrin, 2016). In ML, ensemble learning algorithms combine multiple ML algorithms to obtain a better model. The XGBoost model generates the regression or classification trees by taking previous trees and factoring in their predictions to create a new tree to decrease prediction error. Eq. 5 indicates the XGBoost objective function and Eq. 6 shows the model of XGBoost at iteration *j* that needs to be minimized.

$$Y_{i}=F\left(x_{i}\right)=\sum_{d=1}^{D}f_{d}\left(x_{i}\right),f_{d}\in F,i=1,...,n$$ (5)

$$L_{j}=\sum_{\left(i=1\right)}^{n}l\left(y_{i},{\hat{y}}_{i}^{\left(j-1\right)}+f_{j}\left(x_{i}\right)\right)+\Omega \left(f_{j}\right)$$ (6)

where l is a differentiable convex loss function that measures the dissimilarity between the prediction ${\hat{y}}_{i}$ and the target $y_{i}$ . The term Ω penalizes the complexity of the model and it also helps to smooth out the final learned weights to avoid overfitting.

The RF model is an alternative supervised ensemble learning method based on the decision trees (Breiman, 2001), which can also be implemented for regression and classification problems. It is one of the most widely used ML models due to its simplicity in design, high efficiency, less susceptibility to overfitting, handling the noise, and ability to manage a large number of features. The forest is generated by multiple decision trees and each tree possesses the same distribution. The MSE metric is used to solve the regression models. It determines the distance between the nodes to define which branch is better for the forest. The following Eq. 7 describes this concept (Pavlov et al., 2019).

$$y=\sum_{i=1}^{n}\left(\alpha_{i}-\alpha_{i}^{*}\right)k\left(x,x_{i}\right)+b$$ (7)

where *y* is the value of the data point and *n* is the number of samples.

The MLP is the most well-known ANN model that consists of more than one perceptron, which includes a nonlinear activation function. MLP is a supervised learning method containing one or more hidden layers. The training continues until the following equation is minimized.

$$E=\frac{1}{K}\sum_{k=1}^{K}{\left(y_{k}-{\hat{y}}_{k}\right)}^{2}$$ (8)

where $y_{k}$ and ${\hat{y}}_{k}$ are observed and predicted data points, respectively.

Generating an MLP structure is the most important part that significantly influences the performance of the model. It is a prerequisite to defining the number of neurons in each layer and the number of hidden layers during the construction of the model. MLP is often applied to supervised learning problems. The backpropagation method is implemented to tune the weights and biases of the layers (Hesami et al., 2019).

## Results

### *In vitro* Regeneration of Common Bean

Mature embryo explants exposed to 5, 10, and 20 mg L^−1^ BAP for 20 days resulted in enhanced embryo size of approximately 60–70% explants, which allowed to isolate plumular apice explants easily under sterile conditions (Figure 1C). Thereafter, explants were inoculated on a post-treatment medium, which resulted in multiple shoot induction within 2–3 weeks along with callus induction from the basal end of some explants. The explants were cultured in the growth room for 8 weeks to induce multiple shoots (Figure 1D). The analysis of variance exhibited the variable response of input variables (pretreatment, post-treatment, and pretreatment × post-treatment) on *in vitro* regeneration of common bean. Results revealed the significant impact of pretreatment on the regeneration frequency (*p < 0.01*) and shoot length (*p < 0.05*). Results on post-treatment (*p < 0.01*) application of BAP and combination of pretreatment × post-treatment × post-treatment (*p < 0.01*) revealed a significant impact only on the shoot length. On the other hand, shoot counts have remained insignificant to all input variables (Table 1).

**TABLE 1:** Analysis of variance of output variables of common bean.

Treatment	Output variables	*p*-value
**Pretreatment**	Regeneration (%)	0.000^a^
	Shoot counts	0.234
	Shoot length (cm)	0.013*
**Post-treatment**	Regeneration (%)	0.421
	Shoot counts	0.329
	Shoot length (cm)	0.000^a^
**Pretreatment × post-treatment**	Regeneration (%)	0.562
	Shoot counts	0.682
	Shoot length (cm)	0.021*

a *p< 0.01* and *p< 0.05.

**FIGURE 2:**
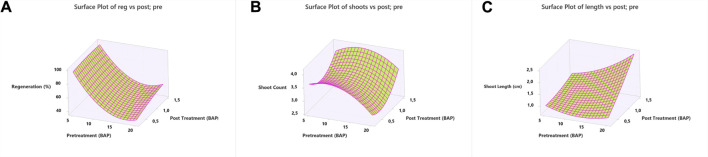
3D response surface plots of *in vitro* regeneration of common bean **(A)** regeneration, **(B)** shoot count, and **(C)** shoot length.

**FIGURE 3:**
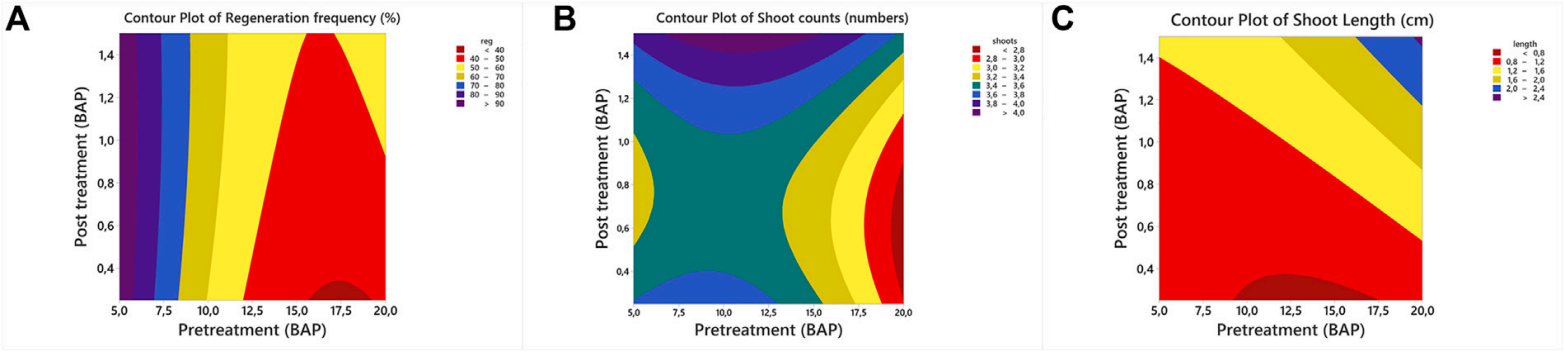
Contour plots of *in vitro* regeneration of common bean **(A)** regeneration, **(B)** shoot count, and **(C)** shoot length.

Results revealed that elevated pretreatment concentrations negatively affected the regeneration frequency and shoot counts, which ranged from 47.91 to 96.51% and 2.99 to 3.60, respectively. In contrast, the mean shoot length increased with elevated pretreatment concentration and ranged from 1.07 to 1.57 cm (Supplementary Table S1). Results of post-treatment revealed a better regeneration frequency at high BAP concentrations ranging from 66.67 to 72.22%. In a similar manner, shoot length also exhibited an increase with a respective increase in BAP concentration. On the other hand, the variable impact of the post-treatment medium (BAP) was observed on shoot counts that ranged from 3.06 to 3.92 (Supplementary Table S1). The results on pretreatment × post-treatment exhibited the negative impact of elevated BAP concentration (pretreatment) on shoot regeneration frequency (%) that ranged from 58.33 to 66.67% (10 m g/L BAP) and 41.67 to 58.33% (20 m g/L BAP). However, exposing explants to 5 mg/L BAP resulted in up to 100% regeneration (Figure 2A). The results on shoot counts and shoot length showed the variable impact of pretreatment × post-treatment concentrations (Figures 2B and C). Outcomes revealed that maximum shoot counts were linked with pretreatment × post-treatment concentrations. The maximum shoot counts were obtained for 10 mg/L × 1.50 mg/L BAP (5.0 shoots), 5 mg/L × 0.50 mg/L BAP (4.67 shoots), and 20 mg/L × 1.50 mg/L BAP (3.33 shoots) (Figure 2B). A similar pattern was also observed with the shoot length. The longest shoots were recorded as 1.15 cm (5 mg L^−1^ × 1.00 mg L^−1^), 1.40 cm (10 mg L^−1^ × 1.50 mg L^−1^), and 1.79 cm (20 mg L^−1^ × 1.50 mg L^−1^) (Supplementary Table S2). Results revealed that exposing explants to a high BAP concentration (both pretreatment and post-treatment) medium yielded relatively longer shoots (Figure 2C) compared to other combinations. In this study, contour plots were also constructed for a better presentation and understanding of the data. In the contour plots, the data were distributed into different subgroups, emphasized with different colors. Contour plots help to find out the best combination for the desired output value. Results of contour plots revealed the optimization of <90% regeneration frequency (Figure 3A), 4.0 shoots per explant (Figure 3B), and 2.4 cm longer shoots (Figure 3C) and presented the doses of the pretreatment and post-treatment medium.

*In vitro* regenerated shoots inoculated on the rooting medium yielded a relatively high rooting frequency. Although most of the plants were rooted within the first 3–4 weeks, they were kept in the rooting medium for a total of 6 weeks before shifting to pots for acclimatization. The survival rate of rooted plantlets in pots was relatively less than expected (Figure 1E). The results revealed that the protocol can be used for *in vitro* regeneration of common bean.

### Response Surface Regression Models

The experiment design of the study was based on pretreatment doses and post-treatment doses, followed by selecting the best mathematical model. The regression equations (Eq. 9–11) for the response variables [*R* ^*2*^ (measured), *R* ^*2*^ (Adj.), and *R* ^*2*^ (pred.)] were used and their respective values are presented in Table 2.

Regeneration frequency = 152.2 − 13.25 pre + 3.9 post + 0.375 pre∗pre − 3.7 post∗post + 0.67 pre∗post (9)

Shoot Counts = 3.52 + 0.124 pre − 1.74 post − 0.00737 pre∗pre + 1.04 post∗post + 0.0263 pre∗post (10)

Shoot Length = 1.511 − 0.117 pre − 0.50 post + 0.00372 pre∗pre + 0.195 post∗post + 0.0710 pre∗post (11)

**TABLE 2:** Response surface regression models for *in vitro* regeneration of common bean.

Output variables	*R* ^*2*^	*R* ^*2*^ adj	*R* ^*2*^ pred
Regeneration (%)	56.27	48.98	38.68
Shoot counts	13.74	0.00	0.00
Shoot Length (cm)	47.84	39.14	24.34

The results of the regression model depicted more *R* ^*2*^ (measured) compared to *R* ^*2*^ (Adj.) and *R* ^*2*^ (pred.) for all tested output variables used in this study. The *R* ^2^ values for regeneration were recorded as 56.27 (*R* ^*2*^ measured), 48.98 (*R* ^*2*^ adj.), and 38.68 (*R* ^*2*^ pred.). *R* ^*2*^ for shoot length was recorded as 47.84 (*R* ^*2*^ measured), 39.14 (*R* ^*2*^ adj), and 24.34 (*R* ^*2*^ pred.). The results illustrated that the regression models efficiently presented the data. In contrast, a low *R* ^*2*^ (measured) value of 13.74 with zero values for both *R* ^*2*^ (adj) and *R* ^*2*^ (pred.) was attributed to the shoot counts. The comparatively low *R* ^*2*^ values for shoot counts reflect that the regression model did not find the association between input and output variables. Overall results illustrated a better impact of pretreatment and post-treatment systems on shoot regeneration and shoot length as compared to shoot counts.

### Response Prediction of Output Variables

**FIGURE 4:**
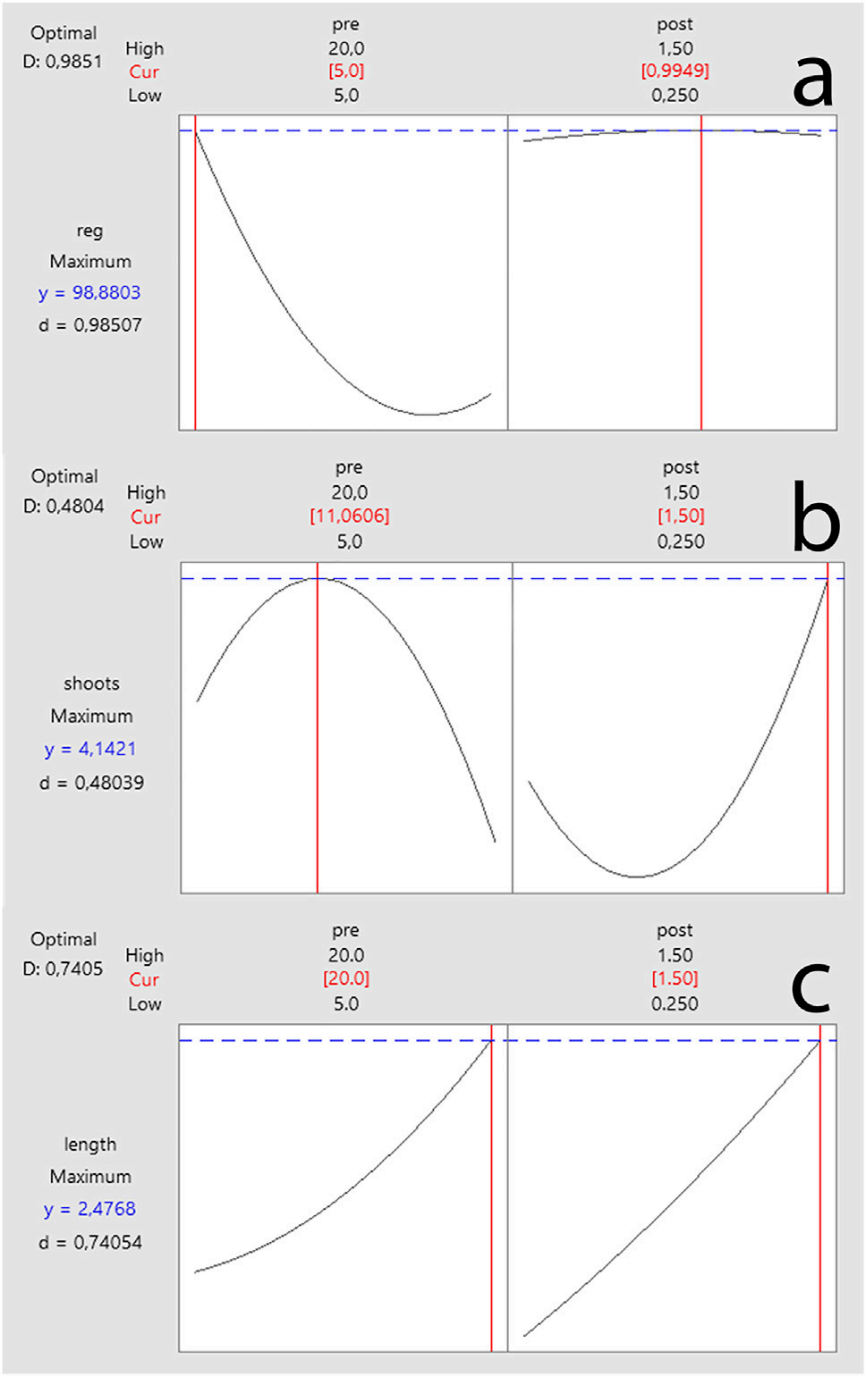
Response prediction of individual output variables on *in vitro* regeneration of common bean **(A)** regeneration, **(B)** shoot count, and **(C)** shoot length.

The computations of predicted values for all output variables of *in vitro* regeneration of common beans were also performed by solving the reference equations (Eq. 7–9) to predict the impact of input variables on output variables. Results indicated a variable combination of pretreatment and post-treatment dose (BAP) for inducing maximum output values. Moreover, results also showed that a combination of 5 mg/L BAP × 0.995 mg/L BAP may yield 98.88% regeneration frequency (%) (Figure 4A). The maximum predicted shoot count (4.142 shoots) was attributed to the 11.061 mg/L BAP × 1.50 mg/L BAP combination (Figure 4B). However, the maximum predicted shoot length of 2.447 cm was attributed to the 20 mg/L BAP × 1.50 mg/L BAP combination (Figure 4C). The predicted values by response prediction models were close to the results attained in this study. The achieved results can be confirmed by checking the contour plots for shoot regeneration frequency (Figure 3A), shoot counts (Figure 3B), and shoot length (Figure 3C).

**FIGURE 5:**
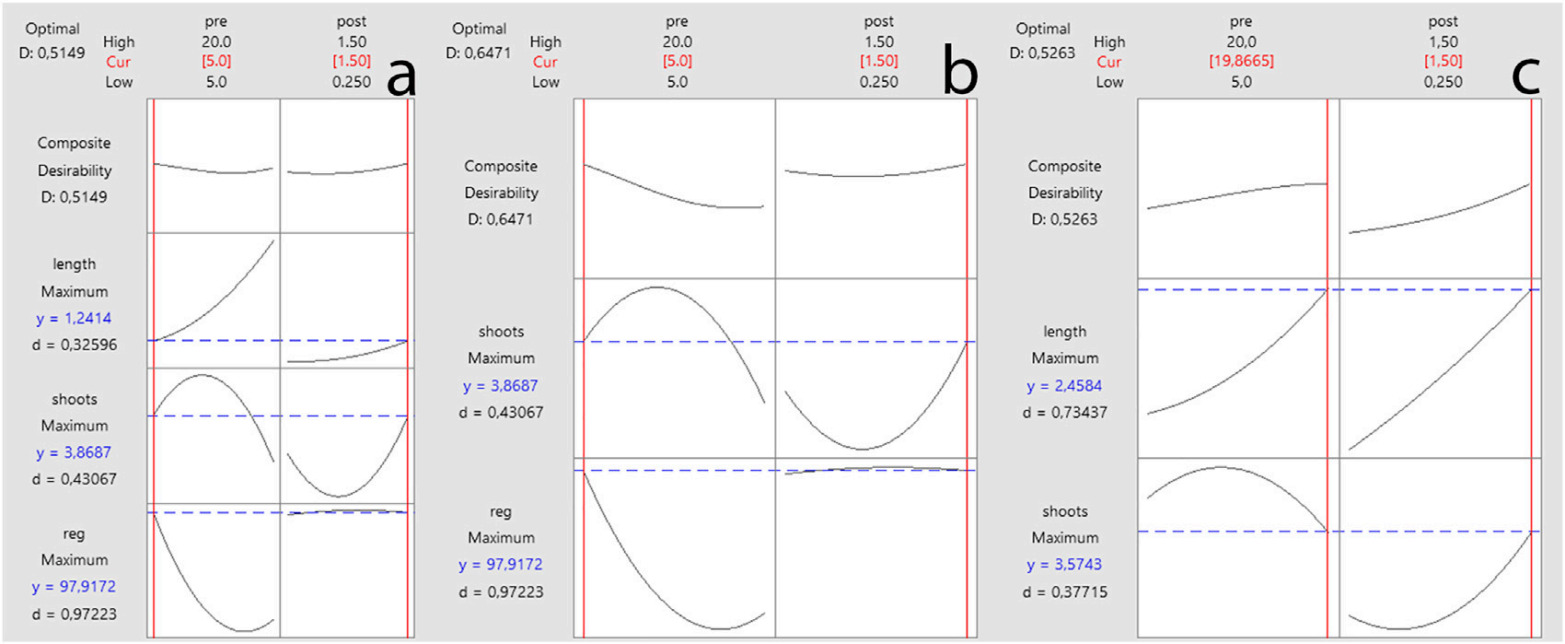
Multiple response prediction of output variables on *in vitro* regeneration of common bean **(A)** regeneration × shoots × length, **(B)** regeneration × shoots, and **(C)** shoots × length.

In addition to response prediction of individual output variables, the prediction response of multiple output variables was also constructed to optimize the dose concentration by considering two (reg × shoots and shoots × length) or considering all output variables (reg × shoot counts × shoot length). Results revealed that the combination of 5 mg/L × 1.5 mg/L BAP can be used for reg × shoot counts × shoot length (Figure 5A) and regeneration × shoot counts (Figure 5B). In contrast, 19.867 mg/L BAP × 1.5 mg/L BAP combination was predicted for shoot counts × shoot length variables (Figure 5C).

### Machine Learning Algorithms

**FIGURE 6:**
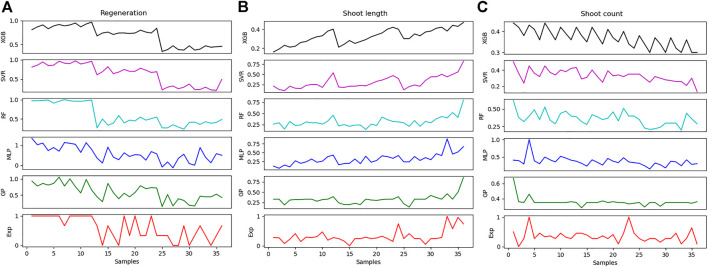
The relationship between the prediction and actual values for **(A)** regeneration, **(B)** shoot length, and **(C)** shoot count.

The *R* ^*2*^ and MSE performance metrics were used to predict the shoot count, shoot length, and regeneration (Table 3). Results exhibited variable *R* ^*2*^ values of all output variables for all models. The MSE values indicate the error between the measured and predicted values and varied with each model. Comparison between the five models revealed the better performance of MLP with high *R* ^*2*^ values for all outputs as compared to other models. However, the values differed for each output variable. Results further illustrated the clear relationship between the *R* ^*2*^ and the MSE values. In general, high *R* ^*2*^ values with low MSE values were recorded for all models. Results on shoot regeneration revealed the order of MLP (0.58 *R* ^*2*^; 0.0596 MSE) > GP (0.49 *R* ^*2*^; 0.0724 MSE) > SVR (0.44 *R* ^*2*^; 0.0802 MSE) > RF (0.44 *R* ^*2*^; 0.0803 MSE) > XGBoost (0.32 *R* ^*2*^; 0.0966 MSE). Results on shoot counts were computed in order of MLP (0.22 *R* ^*2*^; 0.0327 MSE) > SVR (0.11 *R* ^*2*^; 0.0371 MSE) > RF (0.09 *R* ^*2*^; 0.0380 MSE) > GP (0.06 *R* ^*2*^; 0.0392 MSE) > XGBoost (0.01 *R* ^*2*^; 0.0412 MSE). The performance of models on shoot count revealed the order of MLP (0.48 *R* ^*2*^; 0.0258 MSE) > GP (0.35 *R* ^*2*^; 0.0318 MSE) > RF (0.25 *R* ^*2*^; 0.0367 MSE) > SVR (0.23 *R* ^*2*^; 0.0377 MSE) > XGBoost (0.18 *R* ^*2*^; 0.0404 MSE). Figure 6 presents the difference between the predicted and measured values. The horizontal axis refers to the samples while the vertical axis specifies the data collected from the models and the experimental study. The compatibility of the experimental results revealed the better performance of the MLP model for shoot regeneration, shoot counts, and shoot length (Figure 6). On the contrary, the XGBoost model exhibited the least compatibility between actual and predicted values.

**TABLE 3:** Validity of the models.

	Shoot count		Shoot length		Regeneration	
	*R* ^*2*^	MSE	*R^2^*	MSE	*R* ^*2*^	MSE
**MLP**	0.22	0.0327	0.48	0.0258	0.58	0.0596
**SVR**	0.11	0.0371	0.23	0.0377	0.44	0.0803
**GP**	0.06	0.0392	0.35	0.0318	0.49	0.0724
**XGB**	0.01	0.0412	0.18	0.0404	0.32	0.0965
**RF**	0.09	0.0380	0.25	0.0367	0.44	0.0803

**FIGURE 7:**
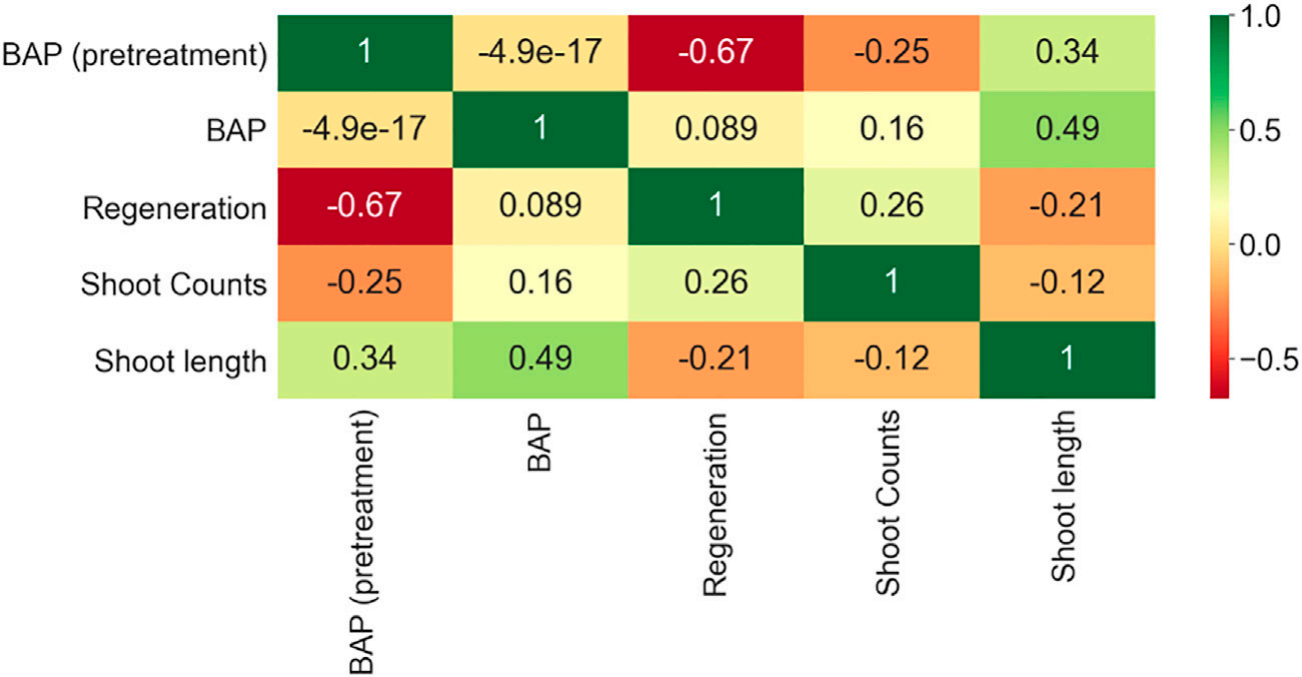
Correlation matrix of inputs and outputs for common bean.

A data visualization method was used with colors to indicate the relationship between two variables. In the heatmap, it was detected that there is a strong correlation between BAP and shoot length. The overall results displayed a negative correlation for regeneration (−0.67) and shoot counts (−0.25) with BAP (pretreatment) and a positive correlation between BAP (pretreatment) and shoot length (0.34). Results on BAP (post-treatment) revealed a positive correlation with all output variables. On the other hand, a negative correlation between regeneration and shoot length (−0.21) and a positive correlation between regeneration and shoot counts (0.49) were also observed (Figure 7). These results indicated the dependence of input factors on output variables.

## Discussion

The selection of proper explants is a prerequisite for establishing an *in vitro* regeneration protocol, especially for recalcitrant plants like edible legumes. The selection of nontraditional and novel explants is one of the possible and potential solutions to overcome the recalcitrant issue in plant tissue cultures (Wang et al., 2011). Plumular apice are a potent and highly efficient explant due to the presence of meristem. To date, researchers tested plumule or plumular apice explant for *in vitro* shoot regeneration of different edible legumes like pea (Molnár et al., 1999), chickpea (Aasim et al., 2013), peanut (Singh and Hazra, 2009; Day and Aasim, 2017), cowpea (Aasim et al., 2009), pigeon pea (Surekha et al., 2005), and lentils (Aasim, 2012). In all these studies, plumular apices were proved to be efficient for inducing high regeneration frequency with high shoot counts per explant. However, the major problem associated with the use of this potent explant is the isolation from the embryo without any damage due to its smaller size. Pretreatment or pulse treatment of mature (Aasim et al., 2009; Day and Aasim, 2017) or immature embryos (Aasim, 2012) with high cytokinin concentration significantly enhances the embryo size which in turn allows isolation of plumular apice explant properly without any damage (Özkan and Aasim, 2019).

Pretreatment of explants with a high dose of cytokinins or auxins for a certain period is more effective to induce more shoots and more rapid regeneration (Brar et al., 1999; Barpete et al., 2014; Kumari et al., 2017; Özkan and Aasim, 2019) due to the more active division of cells (meristematic cells) found in the explants. However, stunted shoots, heavy callus induction, and deformed shoots (vitrified or hyperhidric shoots) are some of the common and negative features associated with the pretreatment (Aasim et al., 2011a). The pretreatment and post-treatment medium, treatment time, plant, and explants (Day and Aasim, 2017) are some of the factors that regulate the whole regeneration process. The manipulation of triggers (inputs), epigenetic and transcriptional cellular responses to the triggers, and molecules stem cell niche (Sugimoto et al., 2019) lead to nondeterministic and nonlinear developmental patterns in the plant’s cells and tissues (Prasad and Gupta, 2008).

*In vitro* regeneration is the mainstay of *in vitro*-based breeding methods, and optimization of input variables for the application of modern biotechnological techniques is highly demanding in the modern era of genome editing. The final output is generally analyzed and interpreted by traditional statistical software programs with the aid of tests like least significant difference test, Duncan’s multiple range test, Tukey’s honestly significant difference test, etc. (Ayuso et al., 2019). These models are not sufficient for the exact prediction of input combinations for the desired output variables. In recent years, modern computer-based software and models have been documented for the exact prediction and validation of the results. The prediction methodologies are divided into three major groups: regression equations, mathematical equations, and computer-based software (Askari et al., 2021). Among these, computer-based software models and simulation programs are gaining popularity with high acceptability by researchers to predict data with more accuracy (Kirtis et al., 2022).

RSM is a computer-based model, used for optimizing and predicting output variables using more than two input variables (Abbasi et al., 2016; Managamuri et al., 2019; Askari et al., 2021; Slimani et al., 2021). The advantage of using contour plots is the distribution of attained results into different subunits, which enables to specify the input variables for the desired output variable (Aasim et al., 2022). RSM predicted the optimal pretreatment and post-treatment BAP concentrations for inducing maximum shoot regeneration frequency, shoot counts and shoot length by estimating the *R* ^*2*^ (measured), *R* ^*2*^ (Adj.), and *R* ^*2*^ (pred.) values of output variables. Furthermore, RSM successfully predicted the input variables by considering individual or multiple output variables. The use of surface plots and contour plots by RSM also clearly illustrated the impact of pretreatment and post-treatment doses of BAP on *in vitro* regeneration output variables of common bean. The use of RSM in plant or agricultural sciences is limited. However, RSM has been employed successfully for predicting the optimal conditions for *in vitro* regeneration and secondary metabolite production of different plants (Bansal et al., 2017; Premkumar et al., 2020; Slimani et al., 2021).

Results on pretreatment dose revealed a negative impact on shoot regeneration frequency and shoot count. Investigation of previous studies on pretreatment with cytokinin discerned the variable impact on shoot regeneration with both positive and negative impacts depending on the genotype, cytokinin type, and concentration. The study on peanuts revealed 100% shoot regeneration with more shoot counts from plumular apices preconditioned with 20 mg L^−1^ BAP as compared to 10 mg L^−1^ BAP (Day and Aasim, 2017). In a similar manner, other studies on cowpea (Brar et al., 1999), *Pongamia pinnata* (Belide et al., 2010), and *Sophora tonkinensis* (Jana et al., 2013) also illustrated the positive impact of pretreatment with cytokinin on shoot regeneration. On the other hand, relatively low regeneration from preconditioned explants has also been documented in peanuts (Akasaka et al., 2000; Matand et al., 2013). The results revealed a decreased shoot count pattern with enhanced pretreatment concentration of BAP. On the contrary, a high concentration of pretreatment dose yielded longer shoots. Results illustrated that shoot counts and shoot length are associated with BAP concentration and other factors like genotype. A previous study on lentils using preconditioned plumular apices explants yielded relatively more shoot counts and shoot length compared to nonconditioned plumular apices explants (Aasim, 2012).

A post-treatment medium enriched with low cytokinin concentration is highly significant and regulates the *in vitro* regeneration from pretreated explants. Results revealed high shoot regeneration frequency, shoot counts, and shoot length from pretreatment and post-treatment of BAP and confirmed the results achieved in chickpea (Aasim et al., 2011b) and lentils (Aasim, 2012). However, the investigation of previous studies clearly illustrated the significance of the correlation between PGRs type and concentration of both pretreatment and post-treatment medium, explant, and genotype (Tang et al., 2012; Kumari et al., 2017) on *in vitro* shoot regeneration. The results confirmed the significance of BAP concentration in the pretreatment and post-treatment medium on *in vitro* shoot count and shoot length of common bean. However, both parameters generated maximum output at a different combination of pretreatment × post-treatment BAP concentration. Previous studies on chickpea and lentils also exhibited a different combination of pretreatment × post-treatment BAP concentration. In chickpea, maximum shoot counts with shorter shoots were associated with high BAP in the post-treatment medium (Aasim et al., 2013). Vice versa, minimum shoot counts with longer shoots of lentils were documented from low BAP concentration in the post-treatment medium (Aasim, 2012). Shoot length is another important factor and maximum shoot length was documented at the high pretreatment × post-treatment combination used in this study. The results are contrary to the findings in peanuts, where shoot length gradually decreased with elevated BAP concentration in the post-treatment medium (Day and Aasim, 2017). Overall results revealed that pretreatment and post-treatment doses of BAP exerted a clear impact on *in vitro* shoot regeneration of common beans as confirmed in other studies (Jahan et al., 2011; Özkan and Aasim, 2019).

*In vitro* rooting of *in vitro* regenerated shoot is an important step to establishing a successful *in vitro* regeneration protocol for recalcitrant plants. The availability of higher cytokinin concentration in the culture medium is generally supposed to be inhibitive for inducing *in vitro* rooting. Previous studies on the use of pretreatment or post-treatment medium in other crops revealed no negative impact on *in vitro* rooting (Aasim et al., 2009; Day and Aasim, 2017; Özkan and Aasim, 2020), and this study also support their findings and achieved 100% rooting. After successful rooting, rooted plants transferred to pots failed to adopt and a very low frequency of plants survived possibly due to awaiting plants in the rooting medium for a long time, which resulted in damaged roots and ultimately affected the survival percentage.

In recent years, ML and ANN models have been successfully employed in plant tissue culture studies for optimizing different input variables like a basal medium (Alanagh et al., 2014; Arab et al., 2016; Arab et al., 2018), PGR types, concentration for *in vitro* regeneration (Kirtis et al., 2022), somatic embryogenesis (Niazian et al., 2017), callogenesis (Niazian et al., 2018), *in vitro* sterilization (Hesami et al., 2019; Aasim et al., 2022), and *in vitro* induced double haploid production (Niazian and Shariatpanahi, 2020). The detailed investigation of these studies revealed the use of different performance metrics like *R* ^2^, MSE, RMSE, MAE, etc. to validate different ML and ANN models. In this study, five different ML models including the ANN model were used for optimizing and predicting the results. Results divulged the variable response of all tested models to the target output variable. The best model for all parameters was found to be MLP. However, the RF model ranked second for shoot regeneration and shoot count, and the GP model for shoot length. The results confirmed the previous findings by researchers in plant tissue culture studies. An investigation of ML models revealed that the prediction of the model is dependent on inputs, target outputs, and the type of model used (Hesami et al., 2019; Salehi et al., 2020, 2021; Kirtis et al., 2022).

The performance of all the tested models was validated by computing *R* ^*2*^ and MSE scores. Relatively high *R* ^*2*^ values for shoot regeneration and low *R* ^*2*^ values were recorded for the shoot length. A detailed investigation of ML models in plant tissue culture studies revealed the variable *R* ^*2*^ values for different output variables like *R* ^*2*^ = 0.94 (Hesami et al., 2019), *R* ^*2*^ = 0.56–0.85 (Salehi et al., 2020), and *R* ^*2*^ = 0.70 (Hesami and Jones, 2021) and 0.98–1.0 (Kirtis et al., 2022). The *R* ^*2*^ values obtained in this study are relatively less than those of the previous findings but still validated the results in an efficient way. A low *R* ^*2*^ does not reflect the poor performance of the experiment, but rather reflects the low compatibility between input and output variables. High *R* ^*2*^ values reflect the high compatibility between the input and output variables, and they are obtained when the difference between the mean of the measured values and the predicted values is bigger than the difference between the actual and predicted values. The single performance metric does not predict or validate the results accurately, and therefore more than two performance metrics are generally considered for ML modeling. MSE is another powerful performance metric that reflects the error between the actual and predicted values. High MSE values depict the high error and vice versa. The results on MSE values for all output variables exhibited very low values for all the tested models, which reflects the low error between the actual and predicted values (Kirtis et al., 2022).

## Conclusion

The development of a successful *in vitro* regeneration protocol for the common bean is extremely crucial for the application of modern biotechnological techniques for its improvement. The developed protocol can be employed for the application of *in vitro* biotechnological techniques like genetic transformation and *in vitro* polyploidy induction for its enhancement. Application of ML and ANN models depicted better performance of the MLP model as compared to other models for better prediction and optimization of all output variables. The results achieved in this study proved that ML models are powerful tools to analyze the data and optimize the complex conditions irrespective of the variable inputs, outputs, and responses of models. The accomplished results can be effectively employed for the prediction and optimization of plant tissue culture protocols used for breeding purposes in the future.

## Data Availability

The original contributions presented in the study are included in the article/Supplementary Material, further inquiries can be directed to the corresponding author.
