# Supplementary material for: Innovation in the Breeding of Common Bean Through a Combined Approach of in vitro Regeneration and Machine Learning Algorithms
Source: Front Genet. 2022 Aug 24;13:897696. doi: 10.3389/fgene.2022.897696 (PMC9451102; doi:10.3389/fgene.2022.897696)
Supplement: Supplementary file 1 [file Table1.docx]

**Supplementary Table 1:** Impact of pretreatment and post treatment medium on in vitro regeneration of common bean

| **Pretreatment medium** | | | |
| --- | --- | --- | --- |
| **BAP**  **(mg L^-1^)** | **Regeneration Frequency (%)** | **Shoot**  **counts** | **Shoot length**  **(cm)** |
| **5** | 97.92±2.60A | 3.60±0.34**^ns^** | 1.07±0.08A |
| **10** | 62.50±8.50B | 3.72±0.37 | 1.06±0.16A |
| **20** | 47.92±5.72B | 2.99±0.24 | 1.57±0.27B |
| **Post treatment medium (BAP)** | | | |
| **0.25** | 66.67±10.21**^ns^** | 3.21±0.29**^ns^** | 0.91±0.12B |
| **0.50** | 66.67±11.78 | 3.54±0.45 | 0.97±0.10AB |
| **1.00** | 72.22±7.73 | 3.06±0.23 | 1.35±0.23AB |
| **1.50** | 72.22±9.72 | 3.92±0.48 | 1.72±0.29A |
