# Supplementary material for: Innovation in the Breeding of Common Bean Through a Combined Approach of in vitro Regeneration and Machine Learning Algorithms
Source: Front Genet. 2022 Aug 24;13:897696. doi: 10.3389/fgene.2022.897696 (PMC9451102; doi:10.3389/fgene.2022.897696)
Supplement: Supplementary file 2 [file Table2.docx]

**Supplementary Table 2**: Impact of pretreatment × post treatment medium on in vitro regeneration of common bean

| **Pre conditioning (BAP mg L^-1^)** | **post treatment**  **(BAP mg L^-1^)** | **Regeneration Frequency (%)** | ***Shoot counts**  **(numbers) NS** | **Shoot length**  **(cm)*** |
| --- | --- | --- | --- | --- |
| 5 | 0.25 | 100.00±0.00**^ns^** | 2.92±0.80**^ns^** | 0.90±0.20B |
|  | 0.50 | 100.00±0.00 | 4.67±1.20 | 1.05±0.24AB |
|  | 1.00 | 91.67±8.33 | 3.28±0.15 | 1.15±0.29 AB |
|  | 1.50 | 100.00±0.00 | 3.42±0.30 | 1.10±0.25 AB |
| 10 | 0.25 | 58.33±16.67 | 3.89±0.11 | 0.67±0.21B |
|  | 0.50 | 58.33±22.05 | 3.17±0.17 | 1.05±0.47AB |
|  | 1.00 | 66.67±16.67 | 2.97±0.51 | 1.10±0.75AB |
|  | 1.50 | 66.67±16.67 | 5.00±1.00 | 1.40±0.55AB |
| 20 | 0.25 | 41.67±8.33 | 2.83±0.17 | 1.17±0.17B |
|  | 0.50 | 41.67±16.67 | 2.78±0.22 | 0.81±0.19AB |
|  | 1.00 | 58.33±8.33 | 3.00±0.58 | 1.79±0.73AB |
|  | 1.50 | 50.00±14.43 | 3.33±0.88 | 2.55±0.33A |
